# Evaluating the role of aldosterone synthesis on adrenal cell fate

**DOI:** 10.3389/fendo.2024.1423027

**Published:** 2024-08-07

**Authors:** Amnani Aminuddin, Morris J. Brown, Elena Aisha Azizan

**Affiliations:** ^1^ Department of Medicine, Faculty of Medicine, Universiti Kebangsaan Malaysia, Kuala Lumpur, Malaysia; ^2^ Endocrine Hypertension, Department of Clinical Pharmacology and Precision Medicine, William Harvey Research Institute, Queen Mary University of London, London, United Kingdom; ^3^ National Institute for Health Research (NIHR) Barts Biomedical Research Centre, Barts and The London School of Medicine and Dentistry, Queen Mary University of London, London, United Kingdom; ^4^ Research Center, Hospital Tunku Ampuan Besar Tuanku Aishah Rohani, Universiti Kebangsaan Malaysia Specialist Children’s Hospital, Kuala Lumpur, Malaysia

**Keywords:** primary aldosteronism, CYP11B2, aldosterone synthesis inhibition, adrenal cell fate, homeostasis of adrenal cortex

## Abstract

Hypertension affects one-third of the adult population worldwide, with primary aldosteronism (PA) accounting for at least 5-10% of these cases. The aldosterone synthase enzyme (CYP11B2) plays a pivotal role in PA manifestation, as increased expression of CYP11B2 leads to excess aldosterone synthesis. Physiological expression of CYP11B2 in humans is normally limited to cells of the adrenal zona glomerulosa under tight homeostatic regulation. In PA, however, there are CYP11B2-positive lesions in the adrenal cortex that autonomously secrete aldosterone, highlighting the dysregulation of adrenal cortex zonation and function as a key aspect of PA pathogenesis. Thus, this review aims to summarize the development of the adrenal glands, the key regulators of adrenal cortex homeostasis, and the dysregulation of this homeostasis. It also discusses the development of CYP11B2 inhibitors for therapeutic use in patients with hypertension, as well as the current knowledge of the effects of CYP11B2 inhibition on adrenal cortex homeostasis and cell fate. Understanding the control of adrenal cell fate may offer valuable insights into both the pathogenesis of PA and the development of alternative treatment approaches for PA.

## 1 Introduction

Hypertension is a chronic yet common medical condition that affects one in three adults aged 30 to 79 worldwide (1). Endocrine hypertension accounts for at least 10% of hypertension cases (2). Primary aldosteronism (PA), characterized by excess aldosterone production by the adrenal glands, is one of the common causes of endocrine hypertension. Frequently, the underlying causes of PA include aldosterone-producing adenomas (APA) and idiopathic hyperaldosteronism (IHA) (3). Aldosterone is normally physiologically synthesized in the zona glomerulosa (ZG) cells of the adrenal cortex. As the rate-limiting enzyme that catalyzes the final steps of aldosterone biosynthesis, aldosterone synthase (CYP11B2), is selectively expressed in the ZG (4). However, in APA and IHA, increased expression and activation of CYP11B2 are commonly observed (5).

Owing to the critical role of CYP11B2 in PA manifestation, research on inhibiting CYP11B2 to suppress aldosterone synthesis has gained much attention (6). In the past few years, several selective CYP11B2 inhibitor drugs have been investigated in clinical trials for treatment of PA (ClinicalTrials.gov). These drugs effectively decreased aldosterone levels without affecting the activity of its closely homologous enzyme, 11β-hydroxylase (CYP11B1), which synthesizes cortisol, a vital hormone for regulating body’s stress responses (7–10).

Despite the anticipated positive treatment outcomes of CYP11B2 inhibitors, the effect of inhibiting CYP11B2 on adrenal cell fate is still understudied. Cell fate determination involving centripetal migration and cell differentiation are crucial for zonation and remodeling of ZG, zona fasciculata (ZF) and zona reticularis (ZR) of the adrenal cortex, thus contributing to the proper function of the adrenal glands (11). Could the suppression of CYP11B2, for example, facilitate the differentiation of ZG cells into ZF cells? Or perhaps could the inhibition of CYP11B2 expression lead to the apoptosis of ZG cells? Understanding the potential consequences or compensatory modulations of the steroidogenesis activity and the remodeling or structural changes of the adrenal cortex is thus of profound importance to corroborate the use of these treatments for PA or hypertension in general.

Primarily focusing on the human system, this review will briefly describe an overview of the adrenal glands development and the key regulators involved in adrenal cortex maintenance to provide a clear understanding of the developmental and lineage progression of cells in the adrenal glands. We further highlight the dysregulation in the cellular turnover or homeostasis of the adrenal cortex that may contribute to the onset of PA and endocrine-related hypertension. Additionally, we discuss the development of therapeutic agents that target CYP11B2 directly, considering the role of this enzyme in the pathology of PA. Drawing from both pre-clinical and clinical studies, we delve into the observed effects of CYP11B2 inhibition on the homeostasis and cellular turnover within the adrenal cortex, which seems to significantly influence adrenocortical zonation and function. We suggest that the mechanisms governing adrenal cell fate may offer valuable insights into both the pathogenesis of PA and the development of alternative treatment approaches for PA.

## 2 Overview of the development of the human adrenal glands

Located at the superior poles of each kidney, the human adult adrenal glands are endocrine glands consisting of two major parts: the adrenal cortex and the adrenal medulla. Each part can be distinctly differentiated by its specific histological structures and biological functions (12). The adrenal cortex is the outer layer of the adrenal glands composed of the ZG, ZF, and ZR. Respectively, each zone is responsible for producing steroid hormones, namely mineralocorticoids, glucocorticoids, and androgenic sex hormones (13). Aldosterone is the main mineralocorticoid produced by the ZG, which is involved in controlling normal electrolyte balance and blood pressure (4). The principal glucocorticoid produced by the ZF is cortisol – a hormone essential for normal metabolic functions and immune responses (14). The ZR, the innermost layer of the adrenal cortex, produces small amounts of sex hormones, specifically dehydroepiandrosterone and androstenedione, which are involved in androgenic activity (15). On the other hand, the adrenal medulla, located at the center of the glands, is responsible for releasing adrenaline and noradrenaline for the fight-or-flight response to various stress factors (16). Prior to evolving into functional adult adrenal glands, the human adrenal glands undergo two crucial stages of development and remodeling – the embryonic and post-natal stages (17, 18).

The embryological origins of mammalian adrenal glands include: 1) the neural crest cells, which give rise to the progenitors of chromaffin cells in the adrenal medulla (16), and 2) the celomic epithelium in the urogenital ridge, which forms the progenitors of the adrenal cortex called the adrenogonadal primordium (AGP) (19). During early gestation, there is a marked increase in expression of steroidogenic factor 1 (Sf-1; nuclear receptor subfamily 5 group A member 1 (NR5A1)), a key regulator for adrenal development and steroidogenesis, in a subset of AGP, leading to the formation of the adrenal fetal zone (FZ) (20). The developing adrenal glands emerge as neural crest cells penetrate the FZ, forming the adrenal medulla at the center of the developing organ (21). Subsequently, mesenchymal cells envelop the developing organ, resulting in the formation of the adrenal capsule (22). The FZ then starts to enlarge, and successively, the adrenal definitive zone (DZ) appears between the adrenal capsule and the FZ (23). The development of the DZ is proposed to be regulated by: 1) NR5A1; 2) the fetal adrenal-specific enhancer (FAdE), the repressor of NR5A1; and 3) the glioma-associated oncogene homolog 1 (Gli1), the activator of hedgehog pathway (20, 23). Later in pregnancy, the DZ expands in size in the fetal adrenal and starts to produce cortisol, marking the development of the ZF of the fetal adrenal cortex.

The maturation of the adrenal cortex begins immediately after birth, whereby the cells within the adrenal FZ start to undergo apoptosis, and the adrenal DZ differentiates into two distinct zones, the ZG and the ZF, under the stimulation of angiotensin II (AngII) and adrenocorticotropic hormone (ACTH) (24). During puberty, the adrenal glands undergo a process called adrenarche, characterized by the increased proliferation of cells that produce adrenal androgens between the ZF and medulla layers. This cell layer makes up the ZR of the adrenal glands, completing the maturation of the adrenal cortex (20, 25).

## 3 Mature adrenal cortex homeostasis

**Figure 1:**
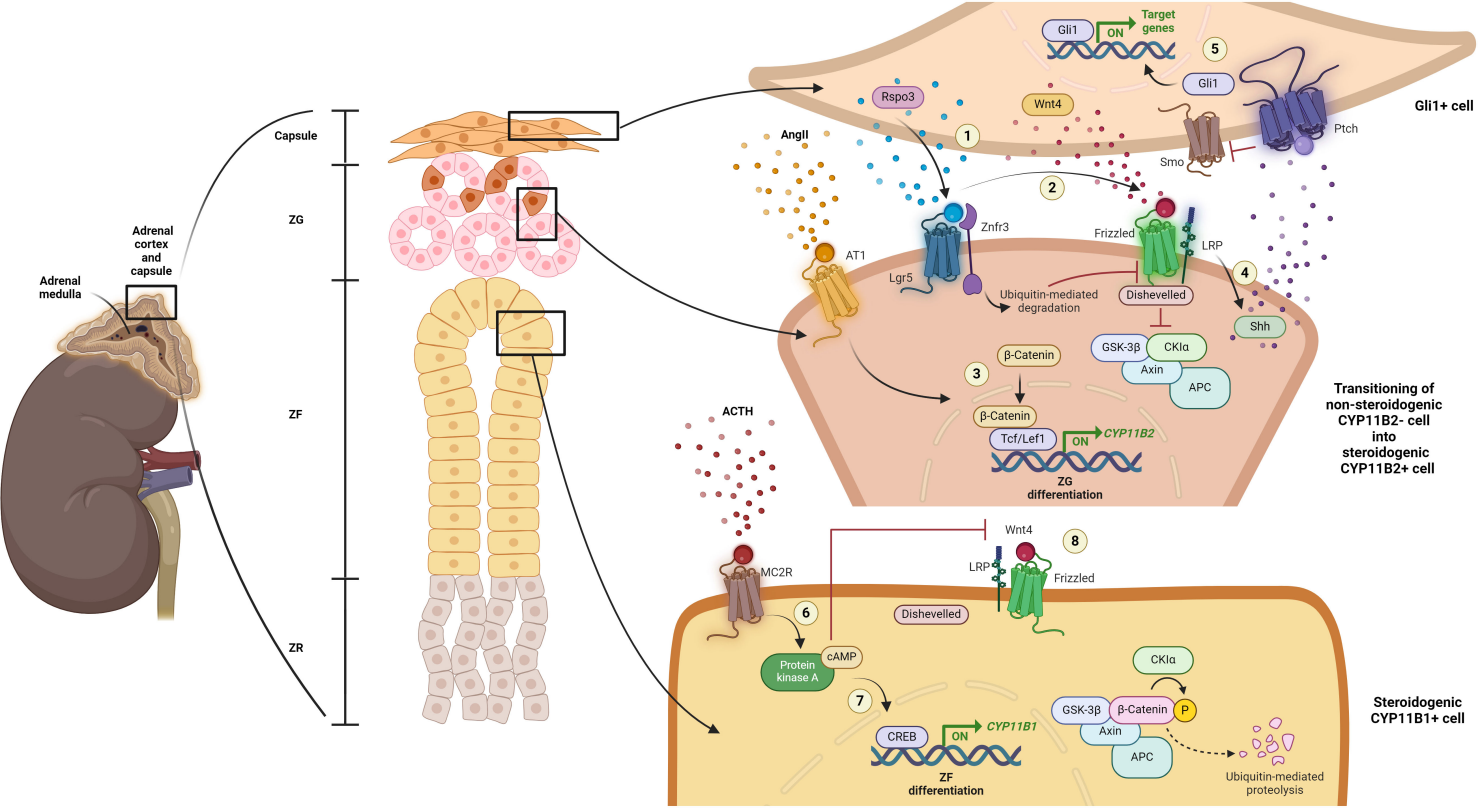
Current understanding of mature adrenal cortex regulation. (1) When in demand, capsular Rspo3 is released and binds to its receptor, leucine-rich repeat-containing G protein-coupled receptor 5 (Lgr5), located within the cells of the adjacent steroidogenic ZG zone, the CYP11B2-negative/Sonic hedgehog protein (Shh)-expressing progenitor cells of the ZG. Simultaneously, Rspo3 binds to the Znrf3 and promotes its ubiquitin-mediated degradation, thereby inhibiting the turnover of the Wnt4 receptor, Frizzled (FZD). (2) This inhibition allows Wnt4 to bind to FZD, promoting further recruitment of Dishevelled. Consequently, β-catenin is accumulated due to the inactivation of the destruction complex comprising adenomatous polyposis coli (APC), AXIN, casein kinase 1 (CK1) and glycogen synthase kinase 3 protein (GSK3 protein). (3) Along with the stimulation by AngII, further nucleus translocation and interaction of β-catenin with transcription factors, including T-cell factor/lymphoid enhancer factor 1 (Tcf/Lef1), lead to the expression of genes essential for adrenal cortex zonation and function, especially *CYP11B2*, initiating ZG differentiation. (4) Wnt signaling activation also further promotes Shh activation. (5) In turn, Shh interacts with Patched (Ptch) and Smoothened (Smo) to activate Gli1-mediated gene transcription in Gli1-positive capsular cells, facilitating their cellular proliferation or recruitment into the steroidogenic lineage. (6) Meanwhile, upon ACTH stimulation through melanocortin 2 receptor (MC2R), the cAMP/PKA/CREB signaling pathway is activated. This activation (7) suppresses canonical Wnt signaling, inhibiting ZG zonation and function, and (8) promotes the transcription of genes that drives the differentiation of ZG into ZF lineage. Created with BioRender.com.

Following the maturation of the adrenal glands, the homeostasis of the adrenal cortex is constantly maintained throughout life in response to physiological demands or hormonal feedback regulation for steroid biosynthesis (**Figure 1**) (17, 23, 25–38). As early as 1883, the ‘Standard Model’ of homeostasis for the mature adrenal cortex was described as the centripetal migration model of adrenocytes (39). According to this model, adrenal cortex cells derive from adrenocortical stem/progenitor cells in the capsule or subcapsular region of the outer layer of the glands and further migrate centripetally while changing their phenotypes from the ZG, to the ZF and the ZR successively. The cells then undergo apoptosis at the boundary layer between the ZR and the adrenal medulla (26, 39). Until now, this model is yet to be challenged, and lineage tracing studies, along with recent trajectory analyses from single-cell transcriptomic studies, continue to support the model of centripetal migration for the maintenance of homeostasis and tissue renewal of the mature adrenal cortex (40–42).

Aside from its role as the key regulator and stimulator of mineralocorticoid secretion, the renin-angiotensin-aldosterone system (RAAS) also directly controls the proliferation of adrenal cortical cells. Physiologically, in response to low blood pressure and volume, activation of the RAAS leads to the secretion of a critical effector, AngII. The binding of AngII to the AngII receptor type 1 (AT1) activates Gq signaling, which further initiates the steroidogenic pathway for CYP11B2 biosynthesis in the ZG for aldosterone production (22–25). An *in vivo* study by McEwan et al. (1996) demonstrated that AngII induction, as well as low sodium intake, resulted in increased uptake of bromodeoxyuridine (BrdU), a marker of cell proliferation, and hypertrophy of the ZG and ZR, indicating proliferation of adrenal cortical cells (43).

Both cellular environment stimulation and the interaction between activated regulatory proteins among cells of different phenotypes and functions within the adrenal cortex play a crucial role in maintaining the fate or homeostasis of the adrenal cells. A crucial mediator that regulates both the development of the fetal adrenal glands and homeostasis of the adult adrenal cortex is canonical Wnt signaling. The Wnt family member 4 (Wnt4) is a well-known component involved in the activation of canonical Wnt signaling in adrenocortical cells (36). Canonical Wnt signaling activity is highly specific within the ZG, either maintaining the pool of adrenocortical progenitor cells or promoting the differentiation of these progenitor cells into functional steroidogenic CYP11B2-expressing cells upon AngII stimulation (17, 23, 25, 26, 28, 30). Suppression of canonical Wnt signaling leads to the inhibition of ZG zonation and functional control, allowing for the differentiation of ZG into ZF lineage (28, 29, 36, 37). A study by Drelon and colleagues demonstrated that upon stimulation by ACTH, the activation of cyclic adenosine monophosphate (cAMP)/protein kinase A (PKA)/cAMP response element-binding protein (CREB) signaling pathway results in the inhibition of Wnt/β-catenin activation through the repression of Wnt4 and promotes lineage conversion towards ZF differentiation (36). Another study demonstrated that mice with Wnt4 deficiency exhibit disorganized ZG and aldosterone suppression (44). Concurringly, the transmembrane E3 ubiquitin ligase, zinc and ring finger (Znrf3), which antagonizes Wnt/β-catenin signaling, also impact adrenal cortex homeostasis (38). Loss of Znrf3 expression in a mouse model was found to promote the expansion of ZF (35).

Another crucial mediator involves the interplay between adrenal capsule cells and adrenocortical cells. R-spondin 3 (Rspo3), expressed in the Gli1-positive adrenal capsule cells, has been demonstrated to be an important component to ensure the replenishment of damaged and lost cells for maintenance of adrenal zonation throughout life (17, 27, 30). Deletion of Rspo3 in mice results in a complete reversal of the anticipated activation of the cell recruitment process, leading to impaired adrenal cortex zonation (27). These findings could possibly explain the observed reduction in adrenal cellular number when induced with Rspo3 or when Lgr5 was knocked down (45). The reduced cell number might result from the absence of crosstalk signaling between capsular cells and progenitor cells, leading to a lack of cell turnover.

## 4 Dysregulation in the homeostasis of adrenal cortex

Dysregulation in the adrenal cortex homeostasis can disrupt the control of adrenal glands function, leading to the manifestation of pathological conditions such as Conn’s syndrome, Cushing syndrome, and virilization (37). To illustrate, adrenal hyperplasia or enlargement of the adrenal cortex, as seen in aldosterone-producing diffuse hyperplasia (APDH) and congenital adrenal hyperplasia, results in excessive aldosterone, cortisol and/or adrenal androgens production (46). APDH, along with other characterized CYP11B2-positive adrenal cortical lesions including carcinoma, adenoma, nodules, micronodules, are the underlying causes of PA (46, 47). In general, these lesions are characterized by the abnormal growth of aldosterone-producing adrenal cortical cells, also known as CYP11B2-positive adrenal cortical neoplasm. They are mostly benign neoplasms excluding the malignant aldosterone-producing adrenal cortical carcinoma (48).

In most cases, the development of the CYP11B2-positive adrenal cortical neoplasm is attributed to somatic mutations in common aldosterone-driver genes, namely *KCNJ5*, *CACNA1D*, *CTNNB1*, *ATP1A1*, and *ATP2B3* (49–54). In general (except for *CTNNB1*), these mutations affect the regulation of intracellular calcium concentration, leading to the activation of the CYP11B2 biosynthesis and eventually aldosterone production. For example, gain of function mutations in *CACNA1D* produce aberrant L-type voltage-gated calcium channels, Ca_v_1.3, leading to increased calcium entry due to the mutated channel being activated at much lower potential thresholds of membrane depolarization (49, 50). Similarly, increased intracellular calcium levels also can be caused by the mutated P-type ATPases pumps, Na+/K+ ATPase α subunit and Ca2+ ATPase, which respectively arise from *ATP1A1* and *ATP2B3* mutations. These mutated ATPases promote elevated sodium and calcium permeability, further leading to membrane depolarization and calcium channel opening (49–51). Whereas, mutations in *KCNJ5*, which encodes for the G-protein-activated inward rectifier K+ channel 4 (GIRK4), result in an unselective potassium Kir3.4 channel, leading to increased sodium entry and subsequent cell membrane depolarization and calcium channel opening without the stimulation by AngII (52, 53).

Interestingly, a study by Nanba et al. (2017) found that the prevalence of aldosterone-producing micronodules (APMs), driven by aldosterone-stimulating somatic mutations, is directly proportional to aging (55). They found that the thickness of ZG cells reduced in elderly subjects, corresponding to the suppressed RAAS in older age. Additionally, the expression of CYP11B2 was limited to sporadic micronodules containing mutated genes that cause unregulated aldosterone production. Concurrently, Omata and colleagues demonstrated that the accumulation of computed tomography-undetectable APMs, which mainly harbor *CACNA1D* aldosterone-driver mutations, contributes to the development of idiopathic adrenal hyperplasia (56). This finding challenges the traditional view that idiopathic adrenal hyperplasia results solely from enlargement of the aldosterone-producing zone within the adrenal cortex.

We have previously suggested that the frequency of somatic mutations causing constitutive aldosterone production is due to the selection of cells that are protected from the fate of apoptosis, which we postulate occurs in ZG cells when salt excess suppresses aldosterone synthesis (57). The sharply demarcated, densely stained APMs that have caught the eye since Celso Gomez-Sanchez’ development and sharing of an antisera specific for CYP11B2 (58), contrast strikingly with the complete absence of CYP11B2 in the adjacent ZG which comprises of the endocrine cells whose intended cell fate was to make aldosterone. While it is easy to regard age-related somatic mutation as at best neutral, and sometimes harmful process, the high prevalence of mutations causing APMs – at least 20% of all adults in salt-loving societies [based on prevalence of APMs, and proportion of these in which mutations are found] – suggests a physiological rather than pathological process. Thus, it raises the question of whether APMs could be the life-saving emergency supply of aldosterone, in times of catastrophic loss of sodium/water loss or rises in plasma K^+^.

Meanwhile, the aldosterone-driver mutation in *CTNNB1* that encodes β-catenin, the critical activator of canonical Wnt signaling pathway, is associated with the development of multiple adrenal cortex disorders (54). Activation of Wnt signaling has been found to promote the proliferation of adrenocortical progenitor cells as well as the differentiation of the progenitor cells into ZG cells (59). Several studies reported the genetic predisposition of the mutations is associated with the demographic (52), gender (60), age (61), or pregnancy-related hormonal imbalance factors (62).

## 5 Drug inhibition of aldosterone synthesis

In the early section, we described how abnormal adrenal cortex homeostasis promotes the development of CYP11B2-positive adrenal cortical neoplasms, eventually leading to the manifestation of endocrine-related hypertension. Hence, it is of interest to explore therapeutic agents that directly target CYP11B2-positive adrenal cortical cells to control aldosterone levels, thus reversing endocrine hypertension. Several discoveries of compounds that suppress aldosterone production by targeting the CYP11B2-positive cells have been reported. The compounds that selectively target the CYP11B2-positive cells interrupt either the expression or activity of CYP11B2, and hence are known as aldosterone synthase inhibitors.

LCI699 (Osilodrostat) was the first CYP11B2 inhibitor developed for use in PA and hypertension (63). However, its development was mainly challenged by its poor selectivity for CYP11B2. The drug also showed inhibition on the enzymatic action of its homologous protein, CYP11B1, an enzyme that catalyzes the final step of cortisol synthesis from the precursor 11-deoxycortisol, leading to impairments in metabolism, immune function, and stress response (64, 65). In early 2020, Osilodrostat has been approved by the European Medicines Agency and the Food and Drug Administration for the treatment of patients with Cushing’s syndrome and Cushing’s disease who are not candidates for pituitary surgery or those who have failed surgery respectively (66, 67).

The active development of the selective CYP11B2 inhibitors with undesired inhibition of CYP11B1 has led to several successes (**Table 1**). Baxdrostat is one of the selective CYP11B2 inhibitors that had completed a phase 2 clinical trial for treatment of patients with treatment-resistant hypertension (7, 68–71). From the finding, the drug lowered serum aldosterone levels without affecting the ACTH-induced change in cortisol in a dose-dependent manner, resulting in significant reduction in both systolic and diastolic blood pressure (7, 71). Another promising selective CYP11B2 inhibitor, namely dexfadrostat phosphate, also known as 5R-fadrozole, had also successfully completed phase 2 clinical trials for treatment in patients with PA (10). The discovery of the off-target CYP11B2 inhibition effect of fadrozole, an approved non-steroidal cytochrome P450 19A1 (CYP19A1) inhibitor for breast cancer management, led to the development of its derivative, 5R-fadrozole (72). Targeting differences in the substrate binding pockets, 5R-fadrozole demonstrated precise inhibitory coordination with the catalytic heme unit of CYP11B2, distinguishing its activity from that against CYP19A1 and CYP11B1 (73).

**Table 1:** List of aldosterone synthase inhibitors or suppressors and their descriptions on the target proteins and mechanism of action, and pre-clinical, clinical trials, or clinical use status.

Compound	Target protein	Mechanism of action	Status of the compound
Osilodrostat (LCI699)	CYP11B1, CYP11B2	Inhibits both enzymatic actions of CYP11B1 and CYP11B2 for catalyzing cortisol and aldosterone synthesis respectively.	• Approved for hypercortisolism in Cushing’s syndrome or disease.
LY3045697	CYP11B2	Selectively targets CYP11B2 with 39-fold inhibition effect over CYP11B1.	• Completed phase 1 clinical trial on healthy volunteers for therapeutic use in patients with hypertension, chronic kidney disease, diabetic nephropathy, primary hyperaldosteronism, or cardiac arrhythmias. (ClinicalTrials.gov ID: NCT01750853**;** NCT01821703)
RO6836191/Baxdrostat (CIN-107)	CYP11B2	Provides selective and competitive blockade of CYP11B2 and inhibit aldosterone production without affecting cortisol level.	• Active phase 3 clinical trial on patients with uncontrolled hypertension on two or more medications and with resistant hypertension. (ClinicalTrials.gov ID: NCT06034743) • Completed phase 2 clinical trial on patients with treatment-resistant hypertension. (ClinicalTrials.gov ID: NCT04519658)
5R-Fadrozole/Dexfadrostat phosphate (DP13)	CYP11B2	Effectively forms a precise inhibitory coordination with the catalytic heme unit of the CYP11B2, thus reducing the aldosterone level. No specific binding observed with CYP11B1 and CYP19A1.	• Completed phase 2 clinical trial on patients with PA. (ClinicalTrials.gov ID: NCT04007406)
MLS101/Lorundrostat	CYP11B2	Selectively binds to CYP11B2 reducing plasma aldosterone and systolic blood pressure, with no observed cortisol insufficiency observed.	• Completed phase 2 clinical trial on patients with uncontrolled hypertension. (ClinicalTrials.gov ID: NCT05001945)
Atractylenolide-I	CYP11B2	Competitively binds to substrate binding site of CYP11B2 against heme, a catalyst for aldosterone synthesis.	• Only pre-clinical testing available.
YM750, Acyl-coenzyme A: Cholesterol acyltransferase (ACAT) inhibitor	ACAT	Suppresses CYP11B2 expression by inhibiting intracellular calcium signaling activated by KCl-stimulated depolarization.	• Only pre-clinical testing available.
Tacrolimus; Calcineurin inhibitor	Calcineurin	Suppresses CYP11B2 expression by inhibiting calcineurin/NFATc4 downstream signaling.	• Only pre-clinical testing available.

Other than that, Lorundrostat, a well-tolerated and highly selective CYP11B2 inhibitor, effectively decreased aldosterone levels and systolic automated office blood pressure in uncontrolled hypertension patients with obesity or suppressed renin in a phase 2 clinical study. *In vitro* analysis revealed that Lorundrostat reduced aldosterone with a selectivity ratio of 374:1 for the inhibition of CYP11B2 compared to CYP11B1 (8, 74, 75). Similarly, another potent CYP11B2 inhibitor, LY3045697, also exhibited high *in vitro* selectivity for CYP11B2 over CYP11B1, with a 39-fold difference. Moreover, the drug also demonstrated a favorable therapeutic index for effects on CYP11B2 over CYP11B1 in a phase 1 clinical study for the dose safety and tolerability on healthy subjects (9).

Pre-clinical studies have also explored the efficacy of small molecules designed to specifically inhibit aldosterone synthesis without affecting other enzymes involved in steroidogenesis. For instances, *in vitro* and *in vivo* investigations have highlighted atractylenolide-I as a potent compound (76). This compound selectively suppressed the activity of CYP11B2 by competitively binding to its substrate binding sites, Ala320 and Cys450, rather than to heme, an essential catalyst for aldosterone production. Similarly, Shimada and colleagues demonstrated that YM750, an acyl-coenzyme A: cholesterol acyltransferase (ACAT) inhibitor, suppressed aldosterone production through inhibition of intracellular calcium signaling activated by potassium chloride (KCl)-stimulated depolarization in an *in vitro* study using H295R human adrenocortical carcinoma cell line (77). They found that the inhibition suppressed the expression of nuclear receptor related 1 (NURR1) and nerve growth factor-induced subfamily B (NGFIB), important transcription factors that regulate CYP11B2 transcription. Thus, small molecules that indirectly affect the CYP11B2 expression and activity may also offer potential benefits for managing PA.

Another study had also reported the inhibition of CYP11B2 expression stimulated by KCl depolarization using the calcineurin inhibitor, tacrolimus (78). In both *in vitro* and *ex vivo* studies using mouse and human adrenal tissues, tacrolimus blocked calcineurin, a subunit for the calcium ion sensor calmodulin, leading to dephosphorylation of nuclear factor of activated T cell, cytoplasmic 4 (NFATc4). Inactivation of NFATc4 led to downstream effects including the direct suppression of *CYP11B2* transcription as well as indirect suppression through inhibition of NURR1 expression.

## 6 The effect of CYP11B2 inhibitors on adrenal cortex homeostasis

Pre-clinical studies and clinical trials have demonstrated a promising clinical effectiveness of CYP11B2 inhibitors. However, there is still a gap in literature regarding the impact of these inhibitors on the homeostasis of adrenocortical cells. It is well-established that the maintenance of adrenal cortex is intricately tied to the physiological requirements of the body. Therefore, the question arises – how do CYP11B2 inhibitors influence the overall maintenance or remodeling of adrenal cortex zonation and function or how does adrenal cortex homeostasis adapt to such changes particularly in regard to the cellular turnover of adrenalocortical cells as proposed by the centripetal migration model of adrenal cortex?

A study involving mice with deleted *CYP11B2* demonstrated that when aldosterone was absent, there was an increase in cellular turnover of ZG cells (79). The increased cellular turnover was characterized by the thickening of the ZG layer and the increase in cells that migrated and underwent apoptosis at the boundary layer between the cortex and medulla. Similar cellular turnover effect was demonstrated in the adrenal tissues from monkeys treated with the CYP11B2 inhibitor, baxdrostat (70). Through immunohistochemistry analysis, baxdrostat treatment on monkeys demonstrated increased apoptosis in a dose-dependent manner. Along with the observed increase in apoptosis, the proliferation rate of ZG cells was also increased, associated with the thickening of the ZG. However, this thickening of ZG coincided with an increase in CYP11B2 expression. To note, the cellular turnover of ZG cells continued during the treatment-free period despite the observed reversibility of CYP11B2 expression.

In adult human adrenal glands, a typical finding is of seemingly discontinuous ZG due to a reduction in ZG cell number and the ZG reaching out to the capsule. This appearance is also seen adjacent to many APAs and might be due to the negative feedback of aldosterone from the APA on adjacent ZG cells. By contrast, the ZG adjacent to APAs with either a *KCNJ5* mutation, or with double mutation of *CTNNB1* and either *GNA11* or *GNAQ*, shows prominent ZG hyperplasia, without expression of CYP11B2 (59, 80). It may be that there are two types of maladaptive response to salt-induced suppression of CYP11B2: one which leads to involution of ZG and selection of APM-forming *CACNA1D-*mutant cells that are protected from apoptosis; the other which leads to ZG hyperplasia (as in the CYP11B2^-/-^ mouse) and selection for mutations that confer a proliferative advantage over adjacent cells. The question whether the gain-of-function mutations driving CYP11B2 autonomy are the same as cause the adenomas has not been settled, and there may indeed be a fine balance between cell growth and death depending on the mutation and/or expression level of the mutated channel (81, 82). Double-mutant APAs may prove the rule, with the only unusual feature being the obligatory pairing of CTNNB1 with GNA11/Q mutations. Or they may prove exceptional, with two mutations required to confer a growth advantage over the gross ZG hyperplasia.

Although our recent preliminary *in vitro* findings revealed that transient silencing of CYP11B2 expression in the HAC15 human adrenocortical carcinoma cell line did not significantly induce cellular apoptosis (83), we observed that CYP11B2 silencing activated stress response mechanisms, including autophagy and mitophagy, potentially facilitating cellular adaptation to CYP11B2 modulation through cellular recycling or initiating cellular death response (84). However, further functional studies are required to confirm whether transient silencing of CYP11B2 activates cellular recycling to promote cell survival, initiates subsequent apoptosis, or triggers necrosis.

## 7 Concluding remarks

In conclusion, exploring CYP11B2 inhibitors presents a promising avenue for managing PA and hypertension. However, comprehending their effects on adrenal cortex homeostasis is crucial for ensuring their safety and efficacy. Current research suggests that these inhibitors can induce changes in cellular turnover within the adrenal cortex, impacting adrenocortical zonation and function. Further investigations are necessary to elucidate the mechanisms underlying these changes and to optimize therapeutic strategies for better outcomes in patients with endocrine-related hypertension.

## Author contributions

AA: Conceptualization, Writing – original draft, Writing – review & editing. MB: Conceptualization, Resources, Supervision, Writing – review & editing. EA: Conceptualization, Funding acquisition, Resources, Supervision, Writing – review & editing.
